# The Impact of Metabolic Health and Obesity on Liver Transplant Candidates and Recipients

**DOI:** 10.3390/life14060685

**Published:** 2024-05-27

**Authors:** Alexander S. Vogel, Rebecca Roediger, Dagny von Ahrens, Brett E. Fortune, Jonathan M. Schwartz, Shalom Frager, Kristina R. Chacko, Clara Y. Tow

**Affiliations:** Montefiore Einstein Center for Liver Transplantation, 111 E 210th Street, Rosenthal 2, New York, NY 10467, USA; alvogel@montefiore.org (A.S.V.); dvonahr@montefiore.org (D.v.A.); bfortune@montefiore.org (B.E.F.); jonschwa@montefiore.org (J.M.S.); shfrager@montefiore.org (S.F.); krchacko@montefiore.org (K.R.C.)

**Keywords:** metabolic dysfunction, fatty liver, liver transplantation, steatotic liver disease

## Abstract

Poor metabolic health and obesity have significant impacts on the outcomes of patients suffering from chronic liver disease, particularly those with metabolic dysfunction-associated steatotic liver disease. Patients with such comorbidities who require liver transplant evaluation for advancing liver disease or liver failure require special consideration due to increased risk of cardiovascular disease, renal dysfunction, sarcopenic obesity, and cancer. Those who have had a history of prior bariatric surgery pose specific anatomical constraints and may also be at increased risk of alcohol use disorder. Pre-operative risk assessment as well as strict control of metabolic risk factors are essential to reduce intra-operative and post-liver transplant complications. As immunosuppressive therapy exacerbates metabolic dysfunction and risk for cancer, post-liver transplant care must focus on balancing the need to prevent rejection and the impact of progressive metabolic dysfunction in this unique, but growing, patient population.

## 1. Introduction

Chronic liver disease (CLD) affects over 100 million people in the United States (US) and causes nearly fifty thousand deaths per year, usually from complications of cirrhosis or liver cancer [1]. As the obesity epidemic worsens in the US, the increasing prevalence of poor metabolic health and obesity among CLD patients has led to more adverse outcomes and an escalating need for liver transplantation [2]. Poor metabolic health, defined as the presence of hypertension, dyslipidemia, hyperglycemia, and/or increased abdominal waist circumference, is associated with increased healthcare spending as well as higher morbidity and mortality [3].

One important medical consequence of poor metabolic health and obesity is the development of a chronic liver disease called metabolic dysfunction-associated steatotic liver disease (MASLD). Recently renamed from non-alcoholic fatty liver disease, MASLD is the accumulation of hepatic steatosis that can progress to advanced liver fibrosis due to the presence of steatosis-induced inflammation (metabolic dysfunction-related steatohepatitis, MASH) [4]. Concurrent with the prevalence of obesity, MASLD is now estimated to affect one in three Americans and is associated with a several-fold increased risk of cardiovascular disease and cancer [5,6]. MASH is also predicted to become the leading indication for liver transplantation (LT) in the US within the next decade [7].

Given the growing impact from these comorbidities, it is imperative to better understand how to manage poor metabolic health and obesity for both LT candidates and recipients as these medical conditions carry significant emphasis on patients’ outcomes. This narrative review will explore the current challenges and management paradigms that liver transplant providers must address when caring for patients suffering from poor metabolic health and obesity while being evaluated for LT, on the transplant waitlist, or after LT.

## 2. Liver Transplant Evaluation and Pre-Transplant Management

MASH cirrhosis makes up approximately 20% of patients listed for LT. Patients with MASH cirrhosis have worse pre-transplant waitlist mortality compared to other types of chronic liver disease [8]. Individuals with MASLD have higher rates of cardiovascular disease, renal disease, metabolic disorders, and older age, all of which impact pre- and post-transplant outcomes. A thorough pre-operative assessment is imperative to optimize patients for the peri-operative period and promote excellent long-term outcomes.

### 2.1. Cardiac Risk Assessment

Atherosclerotic cardiovascular (CV) disease is the leading cause of death in patients with MASLD [9,10]. MASLD is a known but underappreciated independent risk factor for both fatal and non-fatal CV events, even after adjusting for overlapping factors such as diabetes and hypertension [9,11]. Proposed mechanisms for this increased CV risk include dyslipidemia, insulin resistance, visceral/hepatic fat content, endothelial dysfunction, and systemic inflammation [12].

When compared to patients transplanted for alcohol-related cirrhosis, patients with MASH cirrhosis were more likely to have a CV event within 1-year post-LT, even after controlling for age, smoking, sex, diabetes, prior CV disease, and metabolic syndrome (26% for MASH vs. 8% for alcohol-related cirrhosis) [13]. The majority of CV events occurred in the peri-operative period. The nature of LT surgery, with rapid volume shifts, need for massive transfusion, electrolyte disturbances, and reperfusion syndrome, puts significant strain on the CV system [10]. In the peri-operative period, patients are susceptible to heart failure and arrhythmias, while in the long term, immunosuppression can cause progressive worsening of CV disease post-transplant [10].

Initial cardiac evaluation of all LT candidates should include electrocardiogram and echocardiogram, followed by risk stratification based on the individual history. When applicable, medical optimization through smoking cessation, glycemic control, normalization of blood pressure, and use of statins should be implemented. In patients with MASH cirrhosis, assessment of coronary artery disease (CAD) is an important part of this evaluation both due to the high rates of CAD in this population and data suggesting that revascularization may decrease the risk of cardiac events post-transplant [13,14]. Cardiac stress testing with dobutamine stress echocardiography or nuclear stress should be performed in all patients with MASH cirrhosis. It is important to note, however, that the sensitivity and specificity of these tests are affected by the physiological vascular changes seen in cirrhosis, including chronotropic incompetence, marked hypotension, and renal insufficiency [10]. Coronary computed tomography angiography with coronary artery calcium scoring has improved sensitivity and specificity but requires a normal body habitus, limiting its utility in patients with obesity and/or ascites [10].

Patients with abnormal stress testing or considered at elevated risk for CAD should undergo cardiac catheterization. Risks of cardiac catheterization include contrast-induced kidney injury and bleeding. Individuals with >70% stenosis should be considered for revascularization if able to tolerate antiplatelet therapy prior to LT [15]. In those where revascularization is being considered prior to transplant, the main treatment option is percutaneous coronary interventions given the significant morbidity and mortality (adjusted odds ratio of death of 6.9) associated with performing coronary artery bypass grafting in patients with cirrhosis [16].

### 2.2. Evaluation of Renal Disease

Renal dysfunction is common in patients with poor metabolic health, particularly for those with MASH cirrhosis. Many metabolic risk factors common in MASLD, including diabetes, metabolic syndrome, and visceral obesity are also associated with the development of chronic kidney disease (CKD) [17]. As such, a two-fold increase in the prevalence of CKD has been observed in patients with MASLD compared to those without MASLD [18]. Those with MASH have higher rates of CKD compared to those with simple steatosis. Pre-transplant renal dysfunction is a significant predictor of post-transplant renal dysfunction, which can contribute significantly to post-transplant morbidity and mortality [19]. MASLD is now the fastest growing indication for simultaneous liver and kidney transplant [20].

It is important to note that serum creatinine levels may overestimate the glomerular filtration rate in patients with cirrhosis due to sarcopenia, abnormal synthetic function, and hyperbilirubinemia. Cystatin C, a low molecular weight protein not altered in cirrhosis, is a useful tool to properly assess renal function and help better identify appropriate candidates for simultaneous liver and kidney transplant [21].

### 2.3. Sarcopenic Obesity and Frailty

Sarcopenic obesity (SO), a condition in which there is both excess adipose tissue and low muscle mass, is increasingly common [22]. In people over 60 years of age in the US, rates of SO are estimated at 12% of men and 33% of women [23]. SO is associated with both liver fibrosis and CV disease, with high rates observed in patients with cirrhosis awaiting LT [24,25]. Sarcopenia and frailty, defined as a decrease in physiologic reserve with an increased vulnerability to stressors, are closely linked to both pre- and post-transplant mortality [24,26].

Obesity can obscure underlying skeletal muscle wasting, requiring dedicated testing to identify sarcopenia. There are multiple validated scores or objective tests to evaluate patients for sarcopenia and frailty with different testing employed by different centers, including the Skeletal Muscle Index [24]. Strength testing as part of the Liver Frailty Index is often used. Nutritional interventions, such as adding a late evening snack and following a high protein diet, may improve liver function and reduce frailty [27,28].

By identifying and improving frailty indices pre-transplant through the implementation of screening and intervention measures such as intensive nutrition and physical therapy, it is possible to improve both the waitlist and post-transplant outcomes [29,30]. In addition to intensive nutritional interventions, physical activity with aerobic and resistance training is recommended to help optimize endurance, cardiopulmonary fitness, and muscle mass. A smartphone-based fitness app for patients with cirrhosis awaiting liver transplantation has shown promise in helping to track and facilitate increased exercise [31].

### 2.4. Hepatocellular Carcinoma

Hepatocellular carcinoma (HCC), the most common primary liver cancer, is a leading indication for liver transplantation. The global incidence of HCC in patients with MASLD is 0.44 per 1000 person-years and increases to 5.29 per 1000 person-years in those with MASH [32]. Retrospective and prospective data from the US and Europe have reported highly variable rates of non-cirrhotic HCC in those with MASLD, ranging between 4% and 50% [33,34,35,36,37]. Obesity, diabetes, and hypertension, all features of poor metabolic health, have long been established as independent risk factors for HCC [38,39,40,41,42].

Patients with MASLD and HCC are often diagnosed at older ages, with later-stage tumors, and are more likely to have infiltrative tumor involvement [34]. It remains unclear if these findings are due to technical challenges related to ultrasound screening in patients with obesity and/or due to underlying mechanisms of carcinogenesis [43,44]. Cancer treatment strategies for HCC are similar in those with MASLD and/or metabolic disease compared to other etiologies. In the US, patients transplanted for MASH have a lower frequency of HCC compared to other etiologies of liver disease; however, data from Europe have shown the opposite [45,46]. Interestingly, in the US and Europe, the frequency of MASH-related HCC liver transplants has been rapidly rising over the years compared to HCC liver transplants from other underlying liver etiologies [47,48].

Lifestyle modifications and pharmacologic alterations in metabolic risk factors have been shown to reduce HCC risk in patients with MASLD. While weight reduction has not shown particular promise, the Mediterranenan diet and 2.5 hours of intense weekly exercise have demonstrated benefit [49,50]. While not specific to just patients with MASLD, prospective data have supported the benefits of coffee consumption, not related to caffeine, by significantly reducing liver cancer risk [51]. Aspirin, metformin, and statins have similarly shown reductions in liver cancer risk both in MASLD and non-MASLD populations [52,53,54,55].

## 3. Concurrent Alcohol Use in Patients with Obesity and Poor Metabolic Health

The nomenclature related to “steatotic liver disease” (SLD) acknowledges that non-alcoholic fatty liver disease and alcohol-associated liver disease (ALD) are not two distinct entities, but rather part of a dynamic spectrum of SLD, now termed MetALD [4]. Those with MetALD are diagnosed as meeting criteria for MASLD and consume at least moderate amounts of alcohol (daily alcohol consumption of 20–50 g in women or 30–60 g in men; weekly alcohol consumption of 140–350 g in women, 210–420 g in men). MetALD can have a MASLD-predominant vs. ALD-predominant phenotype. Research has shown that those with MetALD have a higher risk for hepatic decompensation compared to their MASLD counterparts [56,57].

Bariatric procedures, particularly Roux-en-Y gastric bypass (RYGB), are a known risk factor for the development of alcohol use disorder (AUD). In 2012, a large prospective, multicenter cohort study known as the Longitudinal Assessment of Bariatric Surgery-2 (LABS-2) demonstrated significant increase in the prevalence of AUD following RYGB compared to pre-operative behaviors [58]. Similar findings have been noted in multiple prospective studies published since that time including comparisons to non-bariatric surgeries [59,60,61,62]. Those with AUD and bariatric anatomy have an increased prevalence of ALD and more severe hepatic disease manifestation, such as cirrhosis, hepatic encephalopathy, infection, acute-on-chronic liver failure, and death, compared to their non-bariatric counterparts [62,63,64].

Patients with MASLD and/or history of bariatric surgery, particularly those who are undergoing LT evaluation, should be assessed for alcohol consumption and screened for AUD. Simple screening tools, such as the AUDIT score or the more abbreviated version, AUDIT-C, may be utilized to capture high-risk patients [65,66]. Biomarkers for the detection of alcohol consumption, including urine ethyl glucuronide and phoshatidylethanol, can help identify intermittent and chronic alcohol intake, respectively [67]. Patients who are identified as having MetALD should be offered a multidisciplinary approach to help them achieve long-term sobriety in addition to their metabolic health management. Strategies should include a combination of pharmacologic agents, such as naltrexone or acamprosate, and enrollment in an alcohol treatment program prior to (if possible) and certainly after transplant to prevent a return to harmful alcohol use [68].

### 3.1. Obesity and Metabolic Health Management

According to the most recent American Association for the Study of Liver Diseases (AASLD) guidelines on the evaluation of patients for LT from 2013, a body mass index (BMI) over 40 kg/m^2^ should be considered a relative contraindication to transplantation [15]. However, MASLD has since become a leading indication for liver transplantation in the US with a rising number of patients with BMI over 35 kg/m^2^ on the transplant waiting list. Despite concerns about the peri-operative impact of a BMI over 40 kg/m^2^, review of post-transplant data shows that 3-year graft survival is better in patients with a BMI of 40 kg/m^2^ or 45 kg/m^2^ compared to persons over the age of 60 [69,70]. Therefore, in the appropriate patient with obesity, LT is a viable and life-saving option.

### 3.2. Nutrition

Obesity management is a crucial component of the management of MASH; in one study, a >10% weight loss was associated with the resolution of MASH in 90% of patients and regression of fibrosis in 45% [71]. Prior to considering pharmacologic or surgical treatments for obesity, patients should first be counseled on lifestyle modifications that can lead to significant weight loss. When counseling patients on diet, a multidisciplinary approach with the use of a nutritionist is advised. Avoidance of excess calories, refined carbohydrates, and sugar-sweetened drinks can be recommended to all patients [72]. While many different diets have been associated with weight loss in patients with MASLD, none has been shown to be clearly superior. The Mediterranean diet is often recommended due to its known cardiovascular benefits and associations with decreased hepatic steatosis [72,73]. Coffee consumption of three or more glasses daily has also been associated with decreased risk of fibrosis in patients with MASLD [74]. Even in patients who adopt lifestyle modifications that successfully lead to weight loss, weight regain is common, and thus, ongoing counseling and support is necessary [75].

### 3.3. Medical Obesity Management

Considerable interest exists regarding the use of emerging medical treatments aimed at obesity management [24]. Medical obesity management may be a superior option for the patients with obesity prior to undergoing LT as the presence of portal hypertension increases procedural risk and is thought to be a relative contraindication to bariatric surgery [76]. Glucagon-like peptide-1 receptor agonists (GLP-1 RAs) are the medication class that has garnered most focus for obesity management. In addition to weight loss and improved glycemic control, early studies show additional benefits of GLP-1 RAs, including reduction of hepatic steatosis and fibrosis and improved cardiovascular outcomes and mortality [77,78]. While there are concerns surrounding a reduction in lean body mass with the use of these agents, in two recent trials, once weekly semaglutide was shown to lead to weight loss without significant effects on skeletal muscle mass [79,80]. Data on the use of GLP-1 RAs in patients with decompensated cirrhosis and/or LT candidates remain limited.

Alongside pharmacotherapy, behavioral change is recognized as an important part of successful and sustained obesity management due to its effects on adherence to medications, nutritional interventions, physical activity, and pre- and post-surgery optimization efforts [81]. These behavioral interventions can take many forms including cognitive behavioral therapy and motivational interviewing, amongst many others [82]. Meta-analyses have shown the effectiveness of behavioral weight management interventions combined with diet and exercise interventions on positive weight change [82,83,84]. It is important to note that training and practice with these techniques is necessary for behavioral interventions to be effective.

### 3.4. Medical Management of Other Metabolic Health Factors

In patients with MASLD, managing other metabolic risk factors is essential. Optimal control of diabetes, hyperlipidemia, and hypertension has shown potential benefits for MASH and clear second-order effects in decreasing the risk of CV disease, a major driver of morbidity and mortality in patients with MASLD/MASH [72]. For glycemic control, most anti-hyperglycemic agents can be used safely in patients with compensated cirrhosis including metformin, pioglitazone, DPP-4 inhibitors (with the exception of vildagliptin), GLP1-Ras, and insulin [85]. Once patients are decompensated, insulin is considered the safest agent. Of the anti-hyperglycemic agents, the GLP1-RAs (including semaglutide) and the GLP1/GIP RAs (tirzepatide) are associated with the most weight loss, suggesting that these may be good agents for patients with MASLD. Statin therapy, while associated with rare cases of drug-induced liver injury, is considered safe in patients with cirrhosis. As statins have a proven benefit in decreasing CV risk and have potential benefits in decreasing the risk of decompensation and hepatocellular carcinoma, use should be considered in patients with MASLD [86,87].

## 4. Interventional Obesity Management: Bariatric Surgery and Endobariatric Procedures

Bariatric procedures are the most effective therapies available for the treatment of class III obesity, formally known as morbid obesity and defined as BMI over 40 or over 35 with obesity-related health complications. Over 262,000 bariatric procedures were performed in the United States in 2021, the most common being sleeve gastrectomy (SG) (58.15%) and RYGB (RYGB) (21.5%) [88]. The SPLENDOR trial demonstrated significant benefit in patients with biopsy-proven MASH with hepatic fibrosis without cirrhosis who underwent bariatric surgery versus nonsurgical care [89]. Countless studies have now shown that bariatric surgery can improve hepatic fibrosis, decrease the risk of serious liver-related outcomes, and resolve MASH [72,90,91]. In a trial looking at patients with MASLD and Child-Pugh A cirrhosis, there were low rates of complications (including decompensation) but with increased safety and decreased mortality in patients undergoing SG compared to RYGB [92]. Data have also supported bariatric procedures in patients with compensated cirrhosis without clinically significant portal hypertension in improving metabolic profiles and potential liver dysfunction [93]. The AGA now supports consideration of bariatric procedures in this unique patient population [93].

There is an increasing number of patients with cirrhosis who are being evaluated for LT who have had prior bariatric procedures. A retrospective cohort of 78 patients evaluated for LT with antecedent bariatric surgery showed higher rates of delisting and/or death on the waiting list, which in part was driven by malnutrition and sarcopenia [94]. Patients with prior bariatric surgery had lower transplantation rates compared to matched controls. From a surgical perspective, patients who have undergone previous bariatric surgery will have increased intra-abdominal adhesions particularly along the left lobe and liver hilum. Anecdotal reports have noted increased risk of delayed post-operative return of GI function and oral tolerance of both nutrition and medications. Concerns regarding altered pharmacokinetics and absorption of immunosuppressive drugs in those with RYGB and sleeve gastrectomy have not been supported by small studies [95]. RYGB anatomy poses several post-LT challenges. Duct-to-duct anastomosis may still be performed, but if biliary access is required post-operatively, the limb lengths post-RYGB may prevent endoscopic biliary access and therefore require a surgical or percutaneous approach. Thus, the transplant team would need to discuss these considerations prior to the transplant.

Most liver transplant candidates who have indications for surgical obesity management are unable to undergo bariatric procedures for pre-LT. Most of these patients have underlying portal hypertension leading to decompensated liver disease and thus carry a high risk of post-surgical liver failure and death. Endobariatric procedures, including intragastric balloons and endoscopic sleeve gastroplasty, have also been shown to be effective in leading to weight loss, with some evidence showing reduction in fibrosis. Long-term data regarding the risks and benefits of endobariatrics are lacking, particularly in those with compensated or decompensated cirrhosis [96]. When considering LT, it is imperative to discuss as a multidisciplinary group including the hepatologist and transplant surgeon prior to any anatomical altering procedure, as the goal is to avoid causing barriers to LT.

There is a paucity of data on bariatric surgery after LT. A systematic analysis of nine studies of bariatric surgery post-LT noted highly variable timing of bariatric surgery post-LT (26 months to 6 years) [97]. One case series noted a death 19 months after SG due to multiorgan failure [98]. Another suggested that RYGB may have contributed to the death of a patient, also due to multiorgan failure [99]. Based on these small reports, morbidity and mortality are considered high compared to antecedent and simultaneous LT bariatric surgery.

## 5. Living Donor Liver Transplantation

In concordance with national trends of obesity and MASLD, the proportion of living donor liver transplants (LDLTs) being done for MASH recipients has risen rapidly over the past 10 years from 9.1% in 2011 to 26.2% in 2021, with the highest increase occurring in Hispanic patients with MASH cirrhosis [100]. An important consideration in the recipient with obesity is the graft–recipient weight ratio (GRWR), used in LDLT to determine the appropriate graft volume for the recipient’s metabolic demands. Recipients with obesity may have a limited pool of donors due to the need for a larger donor liver volume. Consideration has been given to using a lower standard GRWR for this population. In one study, recipients with obesity (weight over 100 kg) were more likely to receive a graft with a GRWR under 0.8 (the current standard), but did not experience higher rates of post-operative complications including graft dysfunction, longer length of stay, or 30-day mortality [101].

In a single-center study of LDLT recipients with MASH cirrhosis, recipients had higher intra-operative blood transfusion requirements but no other significant differences in peri-operative complications or post-operative length of stay [102]. Recipients who were declined for LDLT were not rejected due to their metabolic dysfunction or obesity, but rather for donor issues, progression of HCC, or lack of follow up. Given the genetic predispositions for MASH, such as the PNPLA3 polymorphism, there is a theoretically increased risk of recurrent MASLD in the graft of recipients who receive LDLT from a first-degree relative; however, there is not yet sufficient evidence to demonstrate this effect [103,104]. Further prospective work is needed to better elucidate the impact of metabolic health and obesity in LDLT practices.

## 6. Surgical Considerations for Liver Transplantation in Patients with Obesity and Poor Metabolic Health

LT is a complex surgery which may be further complicated by significant visceral adiposity. With the rise in the prevalence of obesity and MASLD, transplant teams are facing the decision of whether to proceed with listing patients with high BMI more frequently than in prior eras. From a feasibility standpoint, the extremely obese may be excluded with programmatic BMI cutoffs, but fat distribution, abdominal domain, herniae, and prior surgery—including bariatric surgery—all must be considered for an individualized approach [105].

Recipients with obesity present challenges throughout the operation from positioning and retractor placement, incision through a thick abdominal wall, and exposure and mobilization of the liver through visceral and retroperitoneal fat dense with varices. An obese abdomen may be cavernous and deep or the recipient fat may substantially limit space for the donor organ, vessels, or conduits to lie appropriately without kinking, making donor organ selection and implantation technique critical. Post-operative monitoring of vessel patency may also be difficult due to poor visualization on ultrasound and potentially necessitate further investigation with CT, angiography, or reoperation [106]. Recipients with obesity are at a substantially higher risk of abdominal wall complications including wound infection or hematoma, fascial dehiscence, and longer term herniae [107].

While there remain many surgical concerns for patients with obesity undergoing LT, the data are mixed on the impact of obesity towards intra-operative complications. Some series describe longer operative times, increased blood loss, longer length of stay, or increased return to the operating room, while others show no difference in blood loss, surgical complications, or difference in patient or graft survival [108,109,110,111,112,113,114]. The Washington University of St. Louis experience showed no difference in operative time, length of stay, or peri-operative complications in a series of 785 livers [112]. Recipients with BMI > 40 did have significantly reduced 5-year graft and patient survival. Another large case series of 813 liver transplants found that patients with obesity had longer case duration and an increased rate of intra-operative technical problems, including hepatic arterial injury or malposition, IVC injury, and uncontrolled bleeding [109]. The University of Colorado found that recipient BMI > 30 was a significant risk factor for return to the operating room, and of those, recipients with more than 20 unit blood loss or with donors reported to drink more than two drinks per day had an 80% rate of return to the operating room typically for evacuation of hematoma [110].

When looking at those with BMI greater than 50 kg/m^2^, LT outcomes are inferior to those with lower BMI ranges. An analysis of US LTs performed between 1988 and 2013 showed that patients with a BMI ≥ 50 had a 1.6-fold risk of death within 30 days, 52% increased risk of graft failure, and 62% risk of mortality; however, studies are unlikely to fully capture the increased complexity of these cases as patients who are deemed “too high-risk” due to obesity are not listed for transplant, leading to an implicit selection bias [115].

## 7. Simultaneous Bariatric Surgery and Liver Transplantation

There is increasing literature to support simultaneous bariatric surgery and LT among selected candidates. Simultaneous bariatric surgery and LT is a potential solution for those with decompensated liver disease who cannot receive antecedent bariatric surgery and fail pre-LT medical obesity management strategies [106,116,117,118,119,120,121,122,123]. SG is preferred over RYGB as the anatomy allows endoscopic biliary access and it is associated with lower risk of leak complications and malnutrition.

The first report of this complex procedure was published in 2013 [106]. Seven patients undergoing simultaneous LT and SG were compared to 37 patients who had successful medical obesity management prior to undergoing LT alone. The average Model for End-Stage Liver Disease (MELD) score, a logarithmic prognostic calculation of 90-day survival in patients with liver failure that is utilized to prioritize patients on the transplant waiting list, at LT was 32 (range 6–40). The mean BMI at LT was 48 kg/m^2^ (range 39–52). Patients who had simultaneous LT and SG had no deaths or graft losses and improved post-LT metabolic profiles compared to their non-SG counterparts. A recent systematic review including four case series of simultaneous LT and SG shows low morbidity and mortality [97].

Technical complications have included staple line leak, hepatic artery thrombosis, bleeding, as well as GI disturbances such as severe reflux, early satiety, excess weight loss, and dysphagia [123]. Staple line leak is a rare but well-described complication of sleeve gastrectomy that could be catastrophic in an immunosuppressed LT patient. Though rare (<1%) after bariatric surgery, splanchnic thrombosis including portal venous thrombosis (PVT) is a well-described complication that could have potentially devastating consequences, including graft loss after LT. This is particularly troublesome in this population of patients already at high risk of PVT due to their portal hypertension and underlying liver disease [124]. Liu et al. reported two patients developing PVT in their experience of combined LT-SG patients [121]. Further investigation is required to understand the risk and potential need for antithrombotic prevention with consideration of early post-operative pharmacologic anticoagulation prophylaxis with potential continuation after hospital discharge. 

Long-term outcomes have also been favorably described with durable weight loss and lower rates of metabolic dysfunction or recurrent MASLD in the graft [123]. One center described a short-interval, staged approach which allowed for a planned return to OR and SG quickly after the LT and ensuring proper liver graft function, but more research is needed to validate this approach [118,125]. Given the highly specialized nature of this combined surgery, transplant centers pursuing simultaneous LT and SG must carefully select appropriate candidates and create a multidisciplinary structure, involving their bariatric surgery team early, to support patients throughout the transplant process.

## 8. Post-Liver Transplant Management of Patients with Poor Metabolic Health

### 8.1. Post-Liver Transplant Morbidity and Mortality

Within the first year post-transplant, patients most commonly face complications due to infection and CVD [46,126]. Recipients with MASH may have prolonged ICU stays due to obesity-related issues such as inability to wean from the ventilator and increasing their risk for infections [127]. Obesity is associated with increased surgical wound and intra-abdominal infections in the first 30 days post-LT [109]. Post-LT diabetes carries an increased risk of both major (e.g., UTI, bacteremia, pneumonia, abdominal abscess) and minor (e.g., cellulitis, oral thrush, surgical site) infections [128]. For patients transplanted for MASLD, one retrospective cohort study found that urogenital infections and surgical wound infections were more common than in those transplanted for other indications [129]. For these reasons, MASH LT recipients have a lower 1-year graft survival compared to LT survival for other etiologies.

CV events are a major cause of mortality, accounting for about one-fourth of all post-LT deaths. Risk factors for cardiovascular events post-LT are associated with pre-existing hypertension, dyslipidemia, and diabetes. Overall, the presence of diabetes was strongly associated with a decrease in 10-year survival [127]. Given this association, patients transplanted for MASH have the highest risk of CV mortality compared to all indications for LT and a 4-times higher chance of a CV event than those transplanted for alcohol-associated liver disease [130]. Statin use improves post-transplant outcomes and is associated with a mortality benefit even as early as 1-year post-transplant [126,131].

Sarcopenic obesity and frailty have a major impact on early post-transplant survival. Sarcopenia is associated with multiple post-transplant complications, notably multiorgan dysfunction, sepsis, respiratory failure, need for surgical reintervention, as well as death. Unfortunately, frailty related to sarcopenia worsens immediately after LT with a nadir around 3 months. Patients typically recover to their pre-transplant baseline by about 6 months, but the majority never recover back to pre-illness weight. In the 40% of patients who can completely recover, they typically had only mild frailty pre-transplant. On average, the improvement in frailty is about 20% above pre-transplant baseline by 1-year post-transplant, and ongoing recovery after the first year is well observed [132]. Structured exercise programs increase muscle strength and exercise tolerance when compared to the standard of care and improve quality of life [133,134].

Long-term post-LT outcomes for MASH are poorer compared to other indications for LT. A 2022 study querying outcomes from the SRTR database found that patients transplanted for MASH have the lowest one-year and three-year survival of all indications for transplant [127]. Ten-year survival is comparable for MASH and alcohol indications, and both are markedly lower than other indications. Ten-year survival for MASH was 67.5% compared to 71–73% for autoimmune and viral etiologies. This relatively poor post-transplant survival in MASH LT recipients is related to the higher incidence of adverse CV events, tied to their metabolic syndrome exacerbated by immunosuppression, pre-transplant frailty that does not fully recover post-transplant, and concomitant risk for renal disease. Malignancy was the second leading cause of death in patients transplanted for MASH [126,135,136]. More prospective work is essential in the transplant field to investigate any promising therapies or immunosuppression practices that can help mitigate these increased risks experienced by this growing population that suffer from poor metabolic health and obesity. Strict adherence to cancer screening guidelines is vital to improving the overall survival of this high-risk population.

### 8.2. Immunosuppression and Poor Metabolic Health

Immunosuppression exacerbates the underlying metabolic dysfunction that existed before LT or leads to de novo metabolic syndrome (Table 1) [137]. Rates of metabolic complications such as diabetes, hypertension, and dyslipidemia increase from approximately 15% pre-LT to over 50% post-LT [138,139].

Calcineurin inhibitors (CNIs), tacrolimus or cyclosporine, serve as the backbone of post-transplant immunosuppression and are taken lifelong. CNIs lead to de novo or exacerbation of pre-existing metabolic dysfunction. Tacrolimus impairs insulin signaling and increases the risk of diabetes and dyslipidemia. Furthermore, CNIs can cause peripheral vasoconstriction especially at the afferent renal arterioles with subsequent risk of hypertension and nephrotoxicity [140]. Corticosteroids, which are used in high doses both immediately following transplantation as well as for the treatment of acute cellular rejection, are associated with diabetes, hypertension, dyslipidemia, and weight gain. Sirolimus contributes to dyslipidemia and is associated with increased CV risk [141].

Given these side effects and the pre-existing high risk of CV disease in the MASLD population, current recommendations are to minimize CNI immunosuppression and early steroid withdrawal. Strict control of metabolic health parameters is essential. Hypertension is typically treated with calcium channel blockers (i.e., amlodipine or nifedipine), in the absence of proteinuria [142]. These agents specifically act on the afferent renal arterioles and counter CNI vasoconstriction. Angiotensin inhibitors or receptor blockers are typically used if proteinuria is present or added approximately one year following LT. However, cautious monitoring of serum potassium levels is needed due to the potassium retention from these drugs. The Swiss Transplant Cohort Study, a nationwide open prospective cohort of patients receiving LT, found that LT recipients with statin exposure after transplant for any diagnosis had overall lower mortality after LT (HR = 0.35; 95% CI 0.12–0.98; *p* = 0.047) and significant reduction of re-transplantation [143]. The benefit of these agents should not be offset by concerns for post-transplant hepatotoxicity or myotoxicity. Optimal control of diabetes with goal Hemoglobin A1C < 7% is recommended according to the current standards set by AASLD and American Society of Transplantation [141].

### 8.3. Chronic Kidney Disease

Chronic kidney disease is a common sequelae of solid organ transplantation. CNIs have been well described to cause kidney injury. A landmark 2003 study found that liver transplantations are more likely to develop CKD within 5 years than any other solid organ transplantation aside from intestinal transplants with 18.1% of patients that underwent liver transplant developing CKD [144]. Diabetes, hypertension, and hepatitis C were risk factors for post-transplant renal failure in addition to age, sex, and race, which were similar across the heart, lung, and liver transplants analyzed. Developing renal failure post-transplant carried a four-fold increased risk of death [144]. Liver recipients who are transplanted for MASLD have an even higher risk due to comorbidities like diabetes and hypertension, which also have deleterious effects on renal function. Up to a third of MASLD transplant patients develop post-transplant CKD compared to 8% for those transplanted for other indications [131].

### 8.4. Recurrent MASLD in the Liver Allograft

Recurrent MASLD post-LT is nearly universal. Up to 60% of patients develop recurrent MASLD within 1-year post-LT [145]. A nearly 90% overall lifetime recurrence of MASLD based on biopsy or transient elastography has been reported [135]. Recurrent MASLD is associated with post-LT weight gain, obesity, hypertension, and dyslipidemia, though the strongest risk factor for recurrent MASLD is diabetes [145,146]. Worse glycemic control correlates with post-transplant steatohepatitis, post-transplant mortality, rejection episodes, and infections [147,148]. There also appears to be an accelerated progression of MASH post-LT, with over 20% of patients already having biopsy proven advanced fibrosis by a median of 4 years post-LT. Despite the recurrence of MASLD and accelerated fibrosis progression, recurrent cirrhosis only accounts for a small proportion (0.3–9.4%) of post-transplant mortality [127,135]. Liver-related death only accounts for 11% of all post-liver transplant deaths with the most common cause being chronic rejection, followed by MASH cirrhosis [126].

## 9. Conclusions and Future Directions

**Figure 1:**
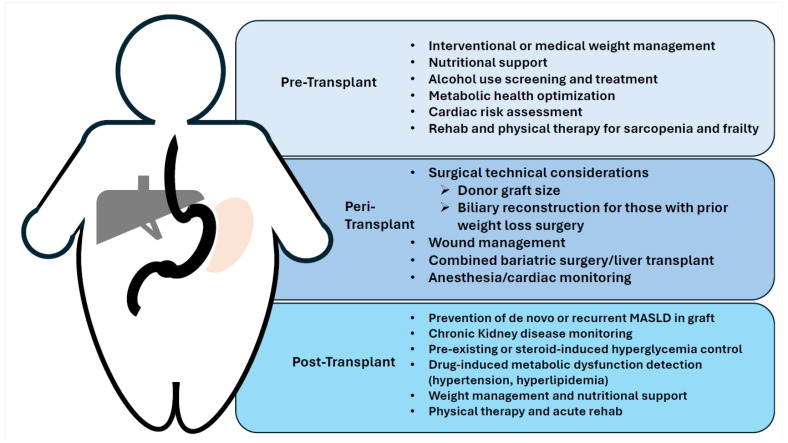
Schematic of simultaneous liver transplant (gray) and sleeve gastrectomy with excised gastric remnant (orange). Clinical considerations in the patient with obesity or post-bariatric liver transplant patient.

Poor metabolic health and obesity are major comorbidities that adversely affect patients with CLD and for those in need or have received LT (Figure 1). While the transplant field continues to expand the utilization of both deceased and living donor organs for recipients with poor metabolic health and obesity, providers must be hypervigilant to augment and control these morbid conditions through all phases of the LT process. Given the significant data that support the adverse impact of metabolic dysfunction on patients, it is essential for providers to address therapeutic strategies, often in multidisciplinary groups, to ensure patients have successful plans for metabolic health control and weight loss prior to transplant (Table 2) and maintaining this regimen well after LT.

More work is needed to improve waitlist and post-LT outcomes for these burdened patients as the demand for LT exponentially increases globally. Additional exploration in weight loss procedures pre-LT, simultaneously, or post-LT will provide promising interventions given the limitations of non-surgical approaches. Furthermore, increasing our understanding of immunosuppression practices and further drug development of new agents with less metabolic toxicity will also assist LT recipients with underlying metabolic dysfunction and/or obesity. However, this effort will require a massive investment and commitment from the field. Multidisciplinary, national, and professional societal efforts as well as government and sponsor funding will be needed to achieve these goals and hopefully translate to improved outcomes for those suffering from CLD along with poor metabolic health and obesity.

## Figures and Tables

**Table 1:** Metabolic impacts of immunosuppressive medications.

Medication Class	Metabolic Impacts	Strategies to Reduce Risk
Calcineurin inhibitors (i.e., tacrolimus, cyclosporine)	Increased risk of diabetes, dyslipidemia, hypertension	Minimize level to achieve normal organ function
Corticosteroids (i.e., prednisone)	Increased risk of diabetes, dyslipidemia, hypertension, weight gain	Early steroid withdrawal
mTOR inhibitors (i.e., sirolimus, everolimus)	Increased risk of dyslipidemia	Minimize level to achieve normal organ function. Avoid in patients with significant hyperlipidemia or high cardiovascular risk

**Table 2:** Current therapies available for the management of obesity and poor metabolic health for patients with MASLD.

Therapy	Examples	Weight Loss Observed	MASH Resolution	Improvement in Fibrosis Observed
Nutrition interventions				
Dietary changes	Mediterranean diet	If >10% weight loss	Yes	Yes
Exercise interventions				
Exercise	Moderate exercise 5 times per week for at least a total of 150 min	Variable	Yes	Potential benefit
Medical therapies				
SGLT-2 inhibitors	Dapagliflozin, Empagliflozin	2–3%	Potential benefit	Unknown
GLP-1 receptor agonists	Semaglutide	13%	Yes	Potential benefit
GLP-1/GIP receptor agonists	Tirzepatide	20.9%	Potential benefit	Unknown
Endoscopic therapies				
Bariatric endoscopy	Endoscopic sleeve gastroplasty, primary obesity surgery endoluminal	14%	Potential benefit	Potential benefit
Surgical interventions				
Bariatric surgery	Sleeve gastrectomy, Roux-en-Y gastric bypass	30%	Yes	Yes

## Abbreviations

Chronic liver disease	CLD
United States	US
Metabolic dysfunction-associated steatotic liver disease	MASLD
Metabolic dysfunction-associated steatohepatitis	MASH
Liver transplantation	LT
Cardiovascular	CV
Coronary artery disease	CAD
Chronic kidney disease	CKD
Steatotic liver disease	SLD
Metabolic dysfunction- and alcohol-associated liver disease	MetALD
Alcohol use disorder	AUD
Alcohol-associated liver disease	ALD
Roux-en-Y gastric bypass	RYGB
American Association for the Study of Liver Diseases	AASLD
Body mass index	BMI
Glucagon-like peptide-1 receptor agonists	GLP-1 RAs
Sleeve gastrectomy	SG
Living donor liver transplants	LDLT
Graft–recipient weight ratio	GRWR
Model for End-Stage Liver Disease	MELD
Portal vein thrombosis	PVT
Calcineurin inhibitors	CNI
